# Incremental Interval Type-2 Fuzzy Clustering of Data Streams using Single Pass Method

**DOI:** 10.3390/s20113210

**Published:** 2020-06-05

**Authors:** Sana Qaiyum, Izzatdin Aziz, Mohd Hilmi Hasan, Asif Irshad Khan, Abdulmohsen Almalawi

**Affiliations:** 1 Center for Research in Data Sciences, Universiti Teknologi PETRONAS, Seri Iskandar 32610, Perak, Malaysia; izzatdin@utp.edu.my (I.A.); mhilmi_hasan@utp.edu.my (M.H.H.); 2 Computer Science Department, Faculty of Computing and Information Technology, King Abdulaziz University, Jeddah 21589, Saudi Arabia; aikhan@kau.edu.sa (A.I.K.); balmalowy@kau.edu.sa (A.A.)

**Keywords:** ant colony optimization, data stream, interval type-2 fuzzy c-means, incremental learning

## Abstract

Data Streams create new challenges for fuzzy clustering algorithms, specifically Interval Type-2 Fuzzy C-Means (IT2FCM). One problem associated with IT2FCM is that it tends to be sensitive to initialization conditions and therefore, fails to return global optima. This problem has been addressed by optimizing IT2FCM using Ant Colony Optimization approach. However, IT2FCM-ACO obtain clusters for the whole dataset which is not suitable for clustering large streaming datasets that may be coming continuously and evolves with time. Thus, the clusters generated will also evolve with time. Additionally, the incoming data may not be available in memory all at once because of its size. Therefore, to encounter the challenges of a large data stream environment we propose improvising IT2FCM-ACO to generate clusters incrementally. The proposed algorithm produces clusters by determining appropriate cluster centers on a certain percentage of available datasets and then the obtained cluster centroids are combined with new incoming data points to generate another set of cluster centers. The process continues until all the data are scanned. The previous data points are released from memory which reduces time and space complexity. Thus, the proposed incremental method produces data partitions comparable to IT2FCM-ACO. The performance of the proposed method is evaluated on large real-life datasets. The results obtained from several fuzzy cluster validity index measures show the enhanced performance of the proposed method over other clustering algorithms. The proposed algorithm also improves upon the run time and produces excellent speed-ups for all datasets.

## 1. Introduction

Today, data streams are prevalent in almost every application in the real world. A data stream is defined as voluminous data coming continuously and most likely evolving over time with unknown dynamics [1]. Some examples of applications related to streaming data are fraud detection, weather monitoring, Internet of Things, and website and network monitoring [2,3,4,5]. In such complex real-world problems, uncertainty is most likely to emerge due to inadequate, incomplete, untrustworthy, vague, and inconsistent data [6]. In general, these different kinds of information deficiencies may bring about different types of uncertainties. Handling uncertainty in an inappropriate manner will lead to wrong conclusions [6]. Thus, data streams are determined by three key attributes: an unbounded sequence of data records, huge volumes of data, and uncertainty associated with data. Therefore, data streams create a new and challenging environment for data mining activities. Clustering is an important data mining activity. It is a process of allocating data points into clusters such that data points in a cluster are more similar to one another compared to other data points in different clusters [7]. Clustering algorithms are grouped together into two categories: hierarchical and partitioning algorithms. For effective clustering of large datasets partitioning, algorithms are preferred over hierarchical algorithms. Among several partitioning algorithms, fuzzy-based clustering techniques are widely used. The Fuzzy C-Means (FCM) has the ability to resolve data uncertainty and can be used as a fuzzy clustering method [8,9]. However, performance of FCM in handling noise and large data uncertainties is very sensitive. Therefore, a more effective method in reducing data uncertainty named as Interval Type-2 Fuzzy C-Means (IT2FCM) had been proposed [10]. Hence, IT2FCM is a suitable clustering technique for handling data streams where uncertainty is most likely to emerge in different forms. 

IT2FCM is a clustering method which is based on objective function (OF) and uses the alternating optimization (AO) method for minimizing OF. AO determines appropriate cluster centers by arbitrarily initializing a membership matrix. This random initialization often leads to IT2FCM getting stuck in local optima and thereby fails to generate suitable cluster centers [11,12,13,14,15]. This issue has been addressed in our previous work by optimizing IT2FCM using ant colony optimization (ACO) [15]. However, IT2FCM-ACO generates clusters over a complete dataset which is not desirable for clustering data streams. Since data streams are likely to evolve with time, the clusters thus generated also vary with respect to time. For example, a large amount of healthcare data may require the user to observe clusters generated over time. Moreover, the volume and the time span of incoming data makes it infeasible to store in memory or disk for further analysis. Therefore, a clustering algorithm is required which is capable of partitioning incoming data continuously while considering memory and time restriction issues. In such situations, an incremental clustering approach is more suitable where the algorithm learns with new incoming data without the need to start from scratch, with limited memory requirement, while achieving good efficiency and accuracy.

Lately, numerous algorithms have been proposed for clustering large and streaming datasets. However, the focus has been primarily on FCM clustering. Commonly, there are two main techniques: distributed clustering and progressive or random sampling approach [16]. Distributed clustering is based on different incremental styles. In distributed clustering, data is divided into chunks and the methods cluster each chunk of data at a time. In the progressive and random sampling-based clustering approach, data is clustered by using a small sample size found by either the progressive or random sampling approach and gradually increases the size until the cluster quality can no longer improve. Examples of these methods include random sampling plus extension FCM (rseFCM) [16], multi-round sampling (MRS) [17,18], single-pass FCM(spFCM) [19], bit-reduced FCM(brFCM) [20], kernel FCM(kFCM) [21,22,23], approximate kernel FCM (akFCM) [24], online FCM(oFCM) [25], okFCM, rsekFCM, spkFCM [26]. Among these algorithms, rseFCM, rsekFCM, akFCM, and MRS are suitable for voluminous data but cannot perform clustering incrementally. On the other hand, spFCM, oFCM, brFCM, kFCM, and their variants process data chunk by chunk. Among the incremental methods, oFCM is least efficient and accurate, brFCM performance suffers for multi-dimensional data i.e., the size of the data increases in terms of the number of variables, kFCM is computationally expensive for large datasets, and spFCM performance reduces at a low sampling rate. However, compared to other algorithms, spFCM achieves similar and/or higher accuracy as FCM and scales efficiently for high-dimensional data. Although spFCM has been applied successfully for speeding up clustering, it is limited to the fuzzy clustering method.

Therefore, for scalable and incremental learning of IT2FCM-ACO, this paper proposes a modified IT2FCM-ACO algorithm. IT2FCM-ACO is improvised by implementing a single-pass approach which will generate clusters in a single pass over data with restricted memory. The proposed algorithm not only improves upon the computational efficiency of IT2FCM-ACO for large datasets but also achieves higher accuracy. Moreover, it performs clustering by processing data incrementally. Therefore, the algorithm handles the challenges of IT2FCM-ACO for uncertain, unbounded, and voluminous data streams. The paper is divided into the following sections: Section One describes related work in detail; Section Three gives an overview on background study of IT2FCM-ACO and the single-pass method; Section Four describes proposed methodology in detail, Section Five, analyses the results obtained by comparing the proposed method spIT2FCM-ACO with IT2FCM-ACO, IT2FCM-AO, and GAIT2FCM by means of certain fuzzy evaluation metrics. The computational efficiency of the algorithm is evaluated with regards to run time and speedup along with the statistical analysis of the proposed algorithm. Section Six presents the conclusion of this research study.

## 2. Related Work

Extensible Fast FCM (eFFCM) [27] is a popular method for clustering large data. It generates clusters on samples obtained through a progressive sampling technique. The size of each subsample is constant. This process continues until the sample satisfies the statistical significance test. The cluster centers obtained for the sample are extended to the entire dataset. eFFCM does not generate fuzzy partitions for the entire dataset, rather it generates partitions for the sample which capture the overall nature of the data. It is, though, a simple and fast technique for clustering large datasets. The sampling technique is not efficient and in certain cases, data reduction is not enough for large data. This issue was handled by Random Sampling Plus Extension FCM (rseFCM) method [16]. It is also a simple, non-iterative clustering technique where samples are generated randomly without replacement. However, it is not incremental, thus, not suitable for data streams, and results generated are often less consistent. 

Multi-Round Sampling FCM (MRSFCM) is another random sampling iterative approach for clustering large data [17,18]. In this method, samples of fixed size are generated randomly and are clustered using FCM. In each iteration, a new sample is generated which is combined with the previous sample and the obtained cluster centers are compared with the prior result. If the result is similar, the algorithm will stop, and the result is extended for the complete dataset. Both rseFCM and MRSFCM are very quick algorithms since they cluster samples of data rather than the complete dataset. However, the size of the sample affects their performance. For small sample sizes, their performance reduces as the error between the cluster locations increases. In rseFCM, no statistical method is used to estimate the optimal size of samples. To overcome this issue, a variant of rseFCM was introduced, called Minimum Sample Estimate Random FCM (MSERFCM), which uses a statistical method to determine the correct sample size [28]. 

Single Pass FCM (spFCM) [19], Online FCM (oFCM) [25] and Bit-Reduced FCM (brFCM) [20] are clustering algorithms based on weighted FCM (wFCM). In FCM, each data object is given equal weighting, but in wFCM, weights are introduced to describe the relative significance of each data object in the clustering solution. A data object associated with a higher weight is more significant in determining the cluster centers. It is proven to be an effective clustering algorithm for both large and multi-dimensional datasets. In addition, compared to FCM it can reach optimal solutions in considerably a smaller number of iterations [16,20]. Its drawback is that its performance reduces at small data size. Additionally, no statistical method is proposed for generating samples. The oFCM algorithm is similar to the spFCM where data samples are generated in a similar manner and are clustered using wFCM. The only difference is that the clustered weighted centers that are generated at the end of each iteration are not added as new data points in the next iteration. Though it is similar to spFCM, its performance is considerably inferior in terms of speedup and quality [27]. brFCM was developed to handle the challenge of clustering large images. In this algorithm, the bin centers are generated for the input data and are clustered using wFCM. The number of data objects in each bin represents weights in wFCM. There are different methods in which bins can be generated. One of the easiest methods is s-bin histograms, where the weights are the number of pixels in each bin and the data are the bin centers. brFCM also does not produce partitions for the complete dataset, however, the results obtained can be easily extended to the entire dataset. For a 1D-MRI image dataset, the brFCM had proven to achieve the best performance in terms of accuracy compared to rseFCM, spFCM, and oFCM [16]. However, if bins for the data are not generated efficiently and accurately it will affect the performance of brFCM. Moreover, generating bins for multi-dimensional datasets creates challenges for the algorithm [16].

Kernel FCM (kFCM) [29] is basically a fuzzy partitioning of large datasets in the kernel induced spaces. The basic idea is to map the input data into high-dimensional space using a kernel map so that it becomes a linear clustering problem. In kFCM the input data is not transformed explicitly but it is simply a representation of dot product of kernel function. The kernel function is basically polynomial and radial basis function. Thus, for a given set of data corresponding kernel matrix is generated. Kernel matrix represents all pairwise dot products of the attributes associated with data in the transformed high-dimensional data space. In the FCM objective function is computed based on the distance between the data points and the cluster centers while in kFCM it is computed as the distance between the data points and cluster centers in the kernel space. The major challenge that exists in kFCM is the selection of appropriate kernel functions and the optimal values of associated parameters. Additionally, the kernel matrix required for storing and computing the entire dataset makes it computationally very expensive. To overcome the issue of computational complexity the kernel distance function was approximated by constraining the space in which the cluster centers exist [21]. The difference-of-convex algorithm (DCA) [30] is another approach that has been considered to solve this issue. It has been applied to kFCM to further improve its computational efficiency and reduce memory requirement. There are several variations to kFCM that has been found in the literature such as Random Sampling and Extension kFCM (rsekFCM), Single Pass kFCM (spkFCM), Online kFCM (okFCM) and Weighted kFCM (wkFCM) [16,26] which follows the same basic step as wFCM, rseFCM, spFCM and oFCM respectively but the objective function is now replaced by weighted kFCM. However, the results obtained by these methods are less accurate than kFCM [16]. 

## 3. Background Study

Section 3.1 and Section 3.2 define type-2 and interval type-2 fuzzy set respectively. Section 3.3 explains the working of ant colony-based optimization of interval type-2 fuzzy c-means and Section 3.4 describes single-pass method. 

### 3.1. Definition of Type-2 Fuzzy Set

A type-2 fuzzy set $\left(\tilde{A}\right)$ is given by type-2 membership function $\left(\mu_{\tilde{A}}\left(x,u\right)\right)$ [31].

(1) $$\tilde{A}=\left\{\left(\left(x,u\right),{\;\mu }_{\tilde{A}}\left(x,u\right)\right)|\;\forall \;x\in X,\forall u\in J_{x}\subseteq \left[0,1\right]\right\}$$

such that $0\leq \mu_{\tilde{A}}\left(x,u\right)\leq 1$

$J_{x}$ is the primary membership of x

(2) $$J_{x}=\left\{\left(x,u\right)|u\in \left[0,1\right],\mu_{\tilde{A}}\left(x,u\right)>0\;\right\}$$

### 3.2. Definition of Interval Type-2 Fuzzy Set

When u∈[0,1] and $\mu_{\tilde{A}}\left(x,u\right)=1$ for x∈X, then $\tilde{A}$ is called an interval type-2 FS (IT2 FS). An IT2 FS is completely described by its Footprint of Uncertainty (FOU) which is defined as the union of primary memberships $\left(J_{x}\right)$ [25].

(3) $$\tilde{A}=1/\mathrm{FOU}\left(\tilde{A}\right)$$

It is an expressive equation that means $\mu_{\tilde{A}}\left(x,u\right)=1$ for $\left(x,u\right)\in FOU\left(\tilde{A}\right)$, where primary membership $\left(J_{x}\right)$ given by (2) in which $\mu_{\tilde{A}}\left(x,u\right)>0$ is replaced by $\mu_{\tilde{A}}\left(x,u\right)=1$.

### 3.3. Ant Colony Optimization of Interval Type-2 Fuzzy C-Means

IT2FCM is a clustering method based on an objective function that assigns a membership degree to each datum belonging to each cluster while determining appropriate cluster centers and membership matrix. In IT2FCM two fuzzifiers *m*_1_ and *m*_2_ are defined to manage a higher level of uncertainty associated with data. Therefore, the OF, membership matrix and cluster centroids are obtained for the two fuzzifiers. In our previous work [15] ACO is introduced to minimize an OF given as follows.

(4) $$J_{m_{1}\;}\left(\tilde{U},\tilde{V}\right)=\sum_{i=1}^{c}\sum_{j=1}^{n}u_{ij}^{m_{1\;}}d_{ij}^{2}\;\mathrm{and}\;J_{m_{2}\;}\left(\tilde{U},\tilde{V}\right)=\sum_{i=1}^{c}\sum_{j=1}^{n}u_{ij}^{m_{2\;}}d_{ij}^{2}$$

where, *m_1_* and *m_2_* denotes different fuzzy degrees (*m_1_*, *m_2_* >1); $u_{ij}$ is the type-reduced membership value of $x_{j}$ data for cluster *i*; $d_{ij}^{2}$ is the Euclidean distance between data point $x_{j}$ and cluster center $v_{i}$; *c* represents number of clusters (2≤c≤n−1); *n* total number of dataset; $\tilde{U}$ denotes type-reduced membership matrix for the data $x_{j}$ belonging to each cluster with membership degree $u_{ij}$; $\tilde{V}$ a type-reduced matrix cluster centers $v_{j}$. 

In IT2FCM-ACO algorithm, each data point $x_{j}\in X$ denotes an ant in the real world and are assigned to each of the c clusters based on membership degree. The distribution of each data point is decided by pheromone matrix $P\in R^{c\times n}$. The lower membership matrix $\left({\underline{u}}_{ij}\right)$ and upper $\left({\bar{u}}_{ij}\right)$ membership matrix is initialized based on lower and upper pheromone matrix $\tilde{P}=\left[\underline{p},\bar{p}\right]$ respectively. This is done by adding Gaussian noise N(0,σ) with variance σ to the normalized matrix $\tilde{P}$ as shown in Equations (5) and (6). The lower and upper membership matrix is updated using Equations (7) and (8) respectively.

(5) $${\underline{u}}_{ij}={\underline{p}}_{ij}/\overset{c}{\underset{k=1}{\sum }}{\underline{p}}_{kj}+N\left(0,\sigma \right)$$

(6) $${\bar{u}}_{ij}={\bar{p}}_{ij}/\overset{c}{\underset{k=1}{\sum }}{\bar{p}}_{kj}+N\left(0,\sigma \right)$$

(7) u_ij=min[1/∑k=1c(dijdik)2(m1−1), 1/∑k=1c(dijdik)2(m2−1)]

(8) u¯ij=max[1/∑k=1c(dijdik)2(m1−1), 1/∑k=1c(dijdik)2(m2−1)]

where, $d_{ij}^{2}=\|x_{j}-v_{i}\|$ is the Euclidean distance between data point $x_{j}$ and cluster centers $v_{i}$ (‖·‖ is the Euclidean norm). The cluster centers are updated using Equation (9) where, $V_{L},V_{R}$ is left and right cluster center, respectively.

(9) $$V=\left[V_{L},V_{R}\right]=\underset{u\left(x_{1}\right)\in J_{x_{1}}}{\sum }\ldots .\underset{u\left(x_{n}\right)\in J_{x_{n}}}{\sum }1/\frac{\sum_{j=1}^{n}{\left(u_{ij}\right)}^{m}x_{j}}{\sum_{j=1}^{n}{\left(u_{ij}\right)}^{m}}$$

The values obtained for membership matrix and cluster centers are of type-2 fuzzy set, therefore they are further type-reduced to obtain type-1 fuzzy set. Next, they are defuzzified to attain corresponding crisp values. Type-reduction and defuzzification steps are followed using the Karnik–Mendel approach [31]. In each iteration OF given by Equation (4) is minimized and its minimum value is obtained. The lower and upper pheromone matrix is updated using the lower and upper values of the membership matrix obtained from (7) and (8) respectively and minimum value of OF. The algorithm proceeds until the maximum iteration is achieved or the stable value of OF is estimated. The lower and upper pheromone matrix is given by (10) and (11) respectively.

(10) $${\underline{p}}_{ij}={\underline{p}}_{ij}\times \left(1-\rho \right)+{\underline{u}}_{ij}/{\left(\tilde{J}-J_{min}+\varepsilon \;\right)}^{\alpha }$$

(11) $${\bar{p}}_{ij}={\bar{p}}_{ij}\times \left(1-\rho \right)+{\bar{u}}_{ij}/{\left(\tilde{J}-J_{min}+\varepsilon \;\right)}^{\alpha }$$

where, ${\underline{p}}_{ij}$ and ${\bar{p}}_{ij}$ denotes lower and upper pheromone matrix respectively; ρ is evaporation rate; ε parameter to avoid division by 0; α parameter that affects the convergence speed; ${\underline{u}}_{ij}$ and ${\bar{u}}_{ij}$ lower and upper membership matrix respectively; $\tilde{J}$ type-reduced value of OF; $J_{min}$ minimum value of OF. The detailed description of the IT2FCM-ACO can be found in [15].

### 3.4. Single Pass Fuzzy C-Means

For clustering large or very large dataset incrementally, in spFCM a certain percentage of dataset is loaded depending on current memory. For instance, if 1% of the data is loaded into memory then we have to scan the entire dataset 100 times. Each data access is called partial data access (PDA). For each PDA, data are clustered into c partitions. However, in single pass technique weights are significant. Thus, the objective function and cluster centroids in FCM is modified to accommodate the effects of weights. However, the adjustment in weights does not affect the convergence attribute of FCM. This is because the weighted data points can be assumed as many singleton data points of similar value. In each PDA new samples of data are stacked in the memory and are grouped together alongside the previous weighted data points. Thus, the final clusters are obtained after all the available data are scanned.

## 4. Proposed Methodology

The single-pass approach to IT2FCM-ACO (spIT2FCM-ACO) is proposed for incremental clustering of large or very large datasets as explained in Algorithm 1 on the next page. In the algorithm rather than loading the complete dataset, some percentage of data is loaded based on existing memory. In each Partial Data Access (PDA) input data are distributed into c clusters using IT2FCM-ACO approach. Similar to spFCM the OF and cluster centroids in IT2FCM-ACO are modified to incorporate the effects of weights. In IT2FCM-ACO the OF as in Equation (4) is improvised and is defined as the constrained optimization for m_1_ and m_2_ given by Equation (12). The IT2FCM-ACO will be referred to as weighted IT2FCM-ACO (wIT2FCM-ACO).

(12) $$J_{m_{1}\;}\left(\tilde{U},\tilde{V}\right)=\sum_{i=1}^{c}\sum_{j=1}^{n}w_{j}u_{ij}^{m_{1\;}}d_{ij}^{2}\;\mathrm{and}\;J_{m_{2}\;}\left(\tilde{U},\tilde{V}\right)=\sum_{i=1}^{c}\sum_{j=1}^{n}w_{j}u_{ij}^{m_{2\;}}d_{ij}^{2}$$

where $w_{j}\in R^{n},\;w_{j}>0$ is a set of weights that describes the significance of each attribute.
**Algorithm 1: single passIT2FCM-ACO**Input: X, c, $t_{max},$ m_1_, m_2_, min_impro, ρ, ε, α, ns, $\underline{w},\;\bar{w}$
Output: *U*, *V*, *J_m_* $z_{max}\in N$ and $z_{max}=$1000, min_impro= 10^−5^ Initialize fuzzifier m_1_ = 1.7, m_2_ = 2.6, $J_{min}=inf,$ ACO parameters: ρ= 0.005, ε=0.01, α= 1.0 Initialize ${\underline{p}}_{ij}=1,\;{\bar{p}}_{ij}i=1,\ldots ,\;c\;and\;j=1,\ldots ,\;n$
00Load X as n_s_ sized samples, $X=\left\{X_{1},\;\ldots \ldots ,\;X_{s}\right\}$
01Initialize ${\underline{w}}_{j}=ones\left(c,n_{s}\right),\;{\bar{w}}_{j},=ones\left(c,n_{s}\right)$02For first PDA since there are no previous c weighted points for $n_{s+c}\;$, c=0.03for l=1 to s  do04 for *z*=1 to $z_{max}$ do05  Repeat06   for *j*=1 to $n_{s+c}$
07    for *i*=1, …., c08     set ${\underline{u}}_{ij}={\underline{p}}_{ij}/\overset{c}{\underset{k=1}{\sum }}{\underline{p}}_{kj}+N\left(0,\sigma \right)$09     if ${\underline{u}}_{ij}<0\;then\;{\underline{u}}_{ij}=0$ end if10     if ${\underline{u}}_{ij}>1\;then\;{\underline{u}}_{ij}=1$ end if11     randomly set ${\bar{u}}_{ij}={\bar{p}}_{ij}/\overset{c}{\underset{k=1}{\sum }}{\bar{p}}_{kj}+N\left(0,\sigma \right)$12     if ${\bar{u}}_{ij}<0\;then\;{\bar{u}}_{ij}=0$ end if13     if ${\bar{u}}_{ij}>1\;then\;{\bar{u}}_{ij}=1$ end if14    end for15    for *i*=1, …., c16     
${\underline{u}}_{ij}={\underline{u}}_{ij}/\overset{c}{\underset{k=1}{\sum }}{\underline{u}}_{kj}$
17     
${\bar{u}}_{ij}={\bar{u}}_{ij}/\overset{c}{\underset{k=1}{\sum }}{\bar{u}}_{kj}$
18    end for19   end for20  until $\overset{n_{s+c}}{\underset{j=1}{\sum }}{\underline{u}}_{ij}>0\;and\;\overset{n_{s+c}}{\underset{j=1}{\sum }}{\bar{u}}_{ij}>0,\forall \;1\leq i\leq c$21compute type-1 fuzzy set of cluster centroids where $V_{L},\;V_{R},\;u_{ij}^{L},\;u_{ij}^{R},\;w_{j}^{L},\;w_{j}^{R}$ recomputed using procedure defined in algorithm 2. 22update $\tilde{U}=\left[{\underline{u}}_{ij},\;{\bar{u}}_{ij}\right]$
23obtain the crisp value of cluster centroids $\tilde{V}=V_{L}+V_{R}/2$
24compute crisp value of fuzzy partition matrix using $u_{ij}=u_{ij}^{L}+u_{ij}^{R}/2$
25type reduce weights $w_{j}=w_{j}^{L}+w_{j}^{R}/2$
26calculate objective function using “Equation (12)”27Type reduce objective function ${\tilde{J}}_{m}=J_{m_{1}}+J_{m_{2}}/2$
28if $\tilde{J}<J_{min}^{m}$ then $J_{min}^{m}=\tilde{J}$ end if29for *i*=1, …., c30  for *j* = 1, …, $n_{s+c}$
31   update pheromone matrices $\tilde{P}$
32   
${\underline{p}}_{ij}=w_{j}{\underline{p}}_{ij}\times \left(1-\rho \right)+{\underline{u}}_{ij}/{\left(\tilde{J}-J_{min}+\varepsilon \right)}^{\alpha }$
33   
${\bar{p}}_{ij}=w_{j}{\bar{p}}_{ij}\times \left(1-\rho \right)+{\bar{u}}_{ij}/{\left(\tilde{J}-J_{min}+\varepsilon \right)}^{\alpha }$
34  end for35 end for36 if *t* > 1, if $\left|\tilde{J}\left(t\right)-\tilde{J}\left(t-1\right)\right|<$min_impro break; end if, end if37end for38compute lower and upper values of weight of c condensed data points39$\underline{w}{’}_{j}=\overset{n_{s+c}}{\underset{j=1}{\sum }}\left({\underline{u}}_{ij}\right)w_{j}\;$, 1≤i≤c, $w_{j}=1\forall \;1\leq j\leq n_{s}$40set ${\underline{w}}_{j}=$
$\underline{w}{’}_{j}$
41$\bar{w}{’}_{j}=\overset{n_{s+c}}{\underset{j=1}{\sum }}\left({\bar{u}}_{ij}\right)w_{j}\;$, 1≤i≤c, $w_{j}=1\forall \;1\leq j\leq n_{s}$42set ${\bar{w}}_{j}=$
$\bar{w}{’}_{j}$
43End

In each subsequent PDAs data are grouped into c clusters along with the previous condensed data points. In each PDA the new data points are clustered together with the previous c weighted data points. They are condensed again into new data points and are grouped with new incoming data in the next PDA. This is referred to as incremental clustering. In spFCM a single weight matrix $\left(w_{j}\right)$ defining the weight of an individual data points are used for evaluating cluster centroids. However, for proposed methodology spIT2FCM-ACO, lower weight $\left({\underline{w}}_{j}\right)$ and upper weight $\left({\bar{w}}_{j}\right)$ matrices are defined. Since weights are relatively important in defining the location of the centers, the data object may have different influence on the left center $\left(V_{L}\right)$ and the right center $\left(V_{R}\right)$ of the cluster.

Suppose *X* is a dataset containing *n* data examples $X=\left\{x_{1},x_{2},\ldots \;,x_{n}\right\}$. First, PDA is generated randomly without replacement by taking a certain percentage of the dataset. Let us suppose from the dataset ‘*s*’ PDAs are obtained, $X=\left\{X_{1},\;X_{2},\;\ldots ,\;X_{s}\right\}$, where, $X_{1}$ is the first PDA, $X_{2}$ is second PDA and so on, each containing *n_s_* data examples. Two cases are used to explain the proposed methodology. In the first case, the first sample of data is taken and clustered using IT2FCM-ACO. In Case 2 the subsequent PDAs are generated and are clustered using IT2FCM-ACO. 

Case 1: $X_{s}$, where s = 1 indicate that the first PDA $\left(X_{1}\right)$ is loaded in the memory and there are no previous weighted data points. The lower and upper weight matrices are initialized to 1. Then, the lower and upper membership matrix is initialized with lower and upper values of the pheromone matrix $\left(\tilde{P}\right)$ given by Equations (5) and (6) respectively. Once the membership matrices are obtained, cluster centroids are updated using (13). In this equation, the weights assigned to the data points are to be estimated for left and right cluster centroids as explained in Algorithm 2.

(13) $$\tilde{V}=\left[V_{L},V_{R}\right]=\left[\frac{\sum_{j=1}^{n_{s}}(w_{j}^{L}){\left(u_{ij}^{L}\right)}^{m}x_{j}}{\sum_{j=1}^{n_{s}}(w_{j}^{L}){\left(u_{ij}^{L}\right)}^{m}},\;\frac{\sum_{j=1}^{n_{s}}(w_{j}^{R}){\left(u_{ij}^{R}\right)}^{m}x_{j}}{\sum_{j=1}^{n_{s}}(w_{j}^{R}){\left(u_{ij}^{R}\right)}^{m}}\right]$$

where $x_{j}\in X_{1},\;w_{j}^{L},\;w_{j}^{R}$ are left and right weights assigned to $V_{L},V_{R}$ respectively.
**Algorithm 2: Type-reducing weighted cluster lefts proposed algorithm**00For arbitrary fuzzifier m01compute lefts $v^{\prime }=\left({v^{\prime }}_{i1},\ldots \ldots ,{v^{\prime }}_{id}\right)$ using $\frac{\sum_{j=1}^{n_{s+c}}w_{j}{\left(u_{ij}\right)}^{m}x_{j}}{\sum_{j=1}^{n_{s+c}}w_{j}{\left(u_{ij}\right)}^{m}}$02where $u_{ij}={\underline{u}}_{ij}+{\bar{u}}_{ij}/2$ and $w_{j}={\underline{w}}_{j}+{\bar{w}}_{j}/2$03sort the *n* data patterns in each of d attributes (*l*=1, …, *d*) in ascending order04compute $V_{R}$05  for all data patterns06find interval index *k*
$\left(1\leq k\leq n_{s+c}-1\right)$ such that $x_{kl}\leq v{’}_{il}\leq x_{\left(k+1\right)l}$07for all data patterns 08    if (j≤k) then09     set primary membership $u_{ij}^{R_{1}}={\underline{u}}_{ij}$ and set weight $w_{j}^{R_{1}}={\underline{w}}_{j},\;\forall \;x_{j}$ else10     set primary membership $\;u_{ij}^{R_{2}}={\bar{u}}_{ij}$ and set weight $w_{j}^{R_{2}}={\bar{w}}_{j},\forall \;x_{j}$11    end if12   end for13$u_{ij}^{R}=\left(u_{ij}^{R_{1}}\cup u_{ij}^{R_{2}}\right)$ and $w_{j}^{R}=\left(w_{j}^{R_{1}}\cup w_{j}^{R_{2}}\right)$14  end for15$V_{L}$ is calculated using the same procedure as above replacing the “if statement” as follows16    for all data patterns 17     if (j≤k) then18      set primary membership $u_{ij}^{L_{1}}={\bar{u}}_{ij}$ and set weight $w_{j}^{L_{1}}={\bar{w}}_{j},\;\forall \;x_{j}$ else19      set primary membership $u_{ij}^{L_{2}}={\underline{u}}_{ij}$ and set weight $w_{j}^{L_{2}}={\underline{w}}_{j},\;\forall \;x_{j}$20    end if21    end for 22$u_{ij}^{L}=\left(u_{ij}^{L_{1}}\cup u_{ij}^{L_{2}}\right)$ and $w_{j}^{L}=\left(w_{j}^{L_{1}}\cup w_{j}^{L_{2}}\right)$
23compute maximum value of $V_{R}$ and $V_{L}$ using equation (13) 

Once values of $V_{L},\;V_{R},\;u_{ij}^{L},\;u_{ij}^{R},\;w_{j}^{L},\;w_{j}^{R}$ are determined using Algorithm 2, they are further defuzzified to obtain their crisp values as shown in Algorithm 1, line 23–25. The obtained defuzified values are used to determine the OF. Subsequently, the lower and upper pheromone matrix is updated as shown at line 32 and 33 respectively. The equations of pheromone matrices are modified to incorporate the influence of weights as shown in (14) and (15).

(14) $${\underline{p}}_{ij}=w_{j}{\underline{p}}_{ij}\times \left(1-\rho \right)+{\underline{u}}_{ij}/{\left(\tilde{J}-J_{min}+\varepsilon \right)}^{\alpha }$$

(15) $${\bar{p}}_{ij}=w_{j}{\bar{p}}_{ij}\times \left(1-\rho \right)+{\bar{u}}_{ij}/{\left(\tilde{J}-J_{min}+\varepsilon \right)}^{\alpha }$$

where, ${\underline{p}}_{ij}$ and ${\bar{p}}_{ij}$ represents lower and upper pheromone matrix respectively; $w_{j}$ is type reduced weight $\left(w_{j}={\underline{w}}_{j}+{\bar{w}}_{j}/2\right)$. If the termination condition is satisfied, the data points are condensed into *c* weighted data points. The lower membership matrix with type-reduced weight is used to update the lower weight while the upper membership matrix updates the upper weight given by (16) and (17) respectively.

(16) $$\underline{w}{’}_{i}=\sum_{j=1}^{n_{s+c}}\left({\underline{u}}_{ij}\right)w_{j}\;,\;1\leq i\leq c$$

(17) $$\bar{w}{’}_{i}=\sum_{j=1}^{n_{s+c}}\left({\bar{u}}_{ij}\right)w_{j}\;,\;1\leq i\leq c$$

where $w_{j}=1\forall \;1\leq j\leq n_{s}$ and the weight of the new *c* data points is given ${\underline{w}}_{j}=\underline{w}{’}_{i}$ and ${\bar{w}}_{j}=\bar{w}{’}_{i},\;\forall \;1\leq i\leq c$. Once the data are clustered into *c* weighted points, the data are released from the memory. These clustered data points are added to the next PDA.

Case 2: s > 1: In this case, all the subsequent PDAs are loaded in the memory and are partitioned into clusters together with previous *c* data points. Thus, in each PDA there are $n_{s+c}$ data points stacked in the memory for generating clusters. The new incoming $n_{s}$ data points are loaded in the memory and are assigned weight 1. The condensed *c* data points are assigned lower and upper weights computed from previous clustering given by (16) and (17) respectively. The new data $n_{s+c}$ are clustered using wIT2FCM-ACO and are again reduced to *c* new weighted data points. The new data points are represented by the *c* cluster centroids ${\tilde{v}}_{i}$ and the weights are computed as shown in (18) and (19).

(18) $$\underline{w}{’}_{i}=\sum_{j=1}^{n_{s+c}}\left({\underline{u}}_{ij}\right){\underline{w}}_{j},\;\forall \;1\leq i\leq c$$

(19) $$\bar{w}{’}_{i}=\sum_{j=1}^{n_{s+c}}\left({\bar{u}}_{ij}\right){\bar{w}}_{j},\;\forall \;1\leq i\leq c$$

where, ${\underline{w}}_{j}=1$ and ${\bar{w}}_{j}=1$ for $n_{s}$ data points and ${\underline{w}}_{j}=\underline{w}{’}_{i}$ and ${\bar{w}}_{j}=\bar{w}{’}_{i}$ for *c* condensed data points obtained from previous clustering. Once the data are clustered into *c* weighted data points, the memory is freed from the data. These clustered data points are again added into the next PDA.

In Algorithm 2 the methodology for obtaining type-reduced cluster centers along-with the type-reduced weights are explained. For any arbitrary fuzzifier *m*, weighted cluster centers are computed as shown at line 01 where $u_{ij}$ is evaluated as the mean of lower and upper membership matrix. Similarly, weight $w_{j}$ is determined as the mean of lower and upper weights. Then the data patterns are sorted for each attribute in an ascending order. To compute the right cluster center $\left(V_{R}\right)$ the values of right membership matrix $\left(u_{ij}^{R}\right)$ and right weight $\left(w_{j}^{R}\right)$ are calculated as well. The index of data (k) is determined where the cluster center obtained from line 01 lies between the two successive data. For all the data patterns whose index is less than *k* lower membership matrix and lower weight is considered otherwise upper membership matrix and upper weight is taken. Once all the data are exhausted, right membership matrix and right weight is determined by taking the union of all the memberships and weights as shown at line 13. Similarly, $V_{L}$ is determined by obtaining left membership matrix and left weight from line 15–22. At line 23, the left and right center is determined using Equation (13).

**Figure 1:**
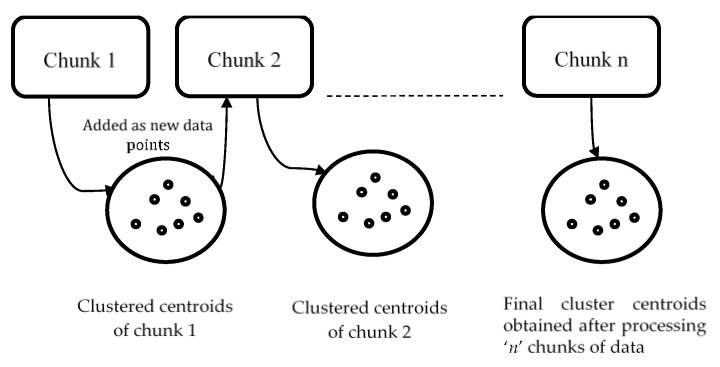
Single Pass clustering approach to IT2FCM-ACO.

Figure 1 shows a single pass clustering technique to IT2FCM-ACO. As shown in figure ‘*n*’ chunks of data are generated randomly from the dataset. Chunk 1 is clustered using IT2FCM-ACO and the obtained cluster centroids are added as new data points to the next data chunk. Chunk 1 is released from the memory, thus, saves time and memory. This process continues until all chunks are scanned and the final clustered data are the obtained cluster centers for the entire dataset.

## 5. Results and Discussion

Section 5.1 gives a brief description of real-world data used in the simulation. Section 5.2 determines the computational complexity of the proposed algorithm. Section 5.3 talks about the simulation results obtained to determine the algorithm performance. It is divided into three Sections: Section 5.3.1 and Section 5.3.2 analyze the results obtained via different fuzzy validity index measures; Section 5.3.3 reports simulation analysis of algorithm efficiency in terms of run time and speedup. Section 5.4 presents the statistical test to validate the performance of the proposed algorithm.

The algorithms are employed in MATLAB R2017a on an Intel® Core™ i7 CPU @ 3.40 GHz with 8GB RAM. During simulation runs the parameters of spIT2FCM-ACO are manually tuned as per our previous study [15] and are summarized in Table 1. The results reported in this section are an average of 10 simulation runs.

### 5.1. Data Description

The datasets used for evaluation of algorithms are classified as large datasets based on Huber classification of dataset [32,33]. Table 2 gives an overview of the dataset used for the experiments. Column 1 represents data name, column 2 gives data size description as per Huber’s classification of data size, columns 3, 4 and 5 presents the number of attributes (#attr), number of examples (#eg) and number of classes (#class) respectively. Typically, six large datasets airlines, forest, sea, poker, electricity, and KDD cup are considered that represents non-stationary real-world data stream problem.

### 5.2. Computational Complexity 

The algorithm efficiency is determined in terms of computational complexity, which is the essential amount of resources required to implement an algorithm. The algorithm computational complexity is determined in terms of run time and space denoted by notation big-O [38]. The following notations are used in the discussion.
*t*maximum iteration over full dataset*t*’average number of iterations over all PDA*n*number of data points*d*dimension of dataset*p*fraction of data loaded in each PDA*s*number of PDAs*c*number of clusters

Time Complexity: In our previous work [15] the time complexity of IT2FCM-ACO was discussed. The time complexity was computed as $O\left(c^{3}ndt\right)$. The computational complexity of spIT2FCM-ACO is determined from Algorithm 1. The first loop at line 03 runs for s number of PDAs and loop at line 04 runs for *t’* iterations i.e., average number of iterations over all PDA. From Line 07–14 the membership matrices are initialized similar to IT2FCM-ACO, and thus, complexity is computed to be $O\left(st’c^{3}nd\right)$. For spIT2FCM-ACO, a segment of data (*p*) are accumulated in each PDA, therefore, the time complexity for clustering each PDA is $O\left(pst’c^{3}nd\right)$. In each PDA a portion of data is acquired in the memory, if 1% of data are loaded the entire dataset must be scanned 100 times. Thus, the number of PDAs is equivalent to the reciprocal of the fraction of data loaded in each PDA (s=1/p). This reduces the computational complexity to $O\left(c^{3}ndt’\right)$. It is found that *t’* compared to *t* is smaller [39]. Thus, spIT2FCM-ACO converges faster than IT2FCM-ACO. The time complexity of IT2FCM-AO is estimated to be $O\left(c^{2}ndt\right)$. Since size of dataset *n* in case spIT2FCM-ACO algorithm is much less than size of complete dataset, thus, convergence rate can be considered faster than IT2FCM-AO. 

Space Complexity: The space complexity of IT2FCM-ACO is O(cn+cd) where *cn* is the size of the membership matrix and *cd* is the size of the dataset. Thus, the space complexity of spIT2FCM-ACO is computed as O(pcn+pcd). Since *p* < *n* the space complexity of spIT2FCM-ACO is less than IT2FCM-ACO. 

### 5.3. Simulation Results for Algorithm Performance

The spIT2FCM-ACO does not produce full data partitions rather work on chunks of data and output final cluster centroids. Therefore, we cannot directly obtain results for different cluster evaluation measures. Thus, the cluster centers obtained at the end of the algorithm run are extended to the entire dataset to generate final clusters. Different fuzzy cluster validity index measures are used to determine the proposed algorithm efficiency and accuracy. Similar to hard cluster partitioning methods [40] we have divided fuzzy cluster validity measures into an internal and external criterion of quality. 

#### 5.3.1. Internal Criterion of Quality

To assess the quality of fuzzy clustering the purpose is to evaluate cluster compactness (the data points in each cluster should be as close as possible) and cluster separation (the clusters themselves should be widely separated). This is an internal criterion for the quality of clustering. In IT2FCM due to premature convergence, the optimal values of cluster centers are not determined and often result in the generation of excess overlapping of clusters. Therefore, both the factors compactness and separation of clusters are measured. 

A validity index proposed by Zahid et al. (SC) [41] is used to evaluate the proposed algorithm. The obtained fuzzy clusters are evaluated based on the attributes of membership degree and format of data to determine fuzzy compactness and fuzzy separation. A higher value of SC indicates well-defined fuzzy partitions. It is computed as follows

(20) $$V_{SC}=SC_{1}\left(c\right)-SC_{2}\left(c\right)$$

where, $SC_{1}\left(c\right)$ considers the geometrical attributes of membership degrees and structure of data and $SC_{2}\left(c\right)$ considers only attributes of membership degree.

(21) $$SC_{1}\left(c\right)=\frac{\sum_{i=1}^{c}\|v_{i}-\bar{v}{\|}^{2}/c}{\sum_{i=1}^{c}\left(\sum_{j=1}^{n}\left(u_{ij}^{m}\right)\|x_{j}-v_{i}\|{}^{2}/\sum_{j=1}^{n}u_{ij}\right)}$$

(22) $$SC_{2}\left(c\right)=\frac{\sum_{i=1}^{c}\sum_{l=i+1}^{n}\left(\sum_{j=1}^{n}{\left(min\left(u_{ij},\;u_{lj}\right)\right)}^{2}\right)/\left(\sum_{j=1}^{n}\left(min\left(u_{ij},\;u_{lj}\right)\right)\right)}{\sum_{j=1}^{n}{\left(\underset{1\leq i\leq c}{\max}u_{ij}\right)}^{2}/\sum_{j=1}^{n}\left(\underset{1\leq i\leq c}{\max}u_{ij}\right)}.\;$$

where, $v_{i}$ center of each cluster, *c* number of clusters, $u_{ij}$ membership degree of data $x_{j}$ to cluster *I*, *m* is fuzzifier value, *n* size of dataset. 

The proposed algorithm spIT2FCM-ACO performance is analyzed and validated against other initialization methods. These include alternating optimization (AO), genetic algorithm (GA) and ant-colony optimization (ACO) without sp incremental approach. Table 3 summarizes the result of SC index for different algorithms along with standard deviation (SD). spIT2FCM-ACO achieves higher values for SC compared to the other three algorithms. Higher values of SC indicate great attachment within clusters and less overlapping between clusters. This suggests the proposed algorithm spIT2FCM-ACO generates optimal cluster centers while improving the clustering quality as compared to other algorithms.

**Figure 2:**
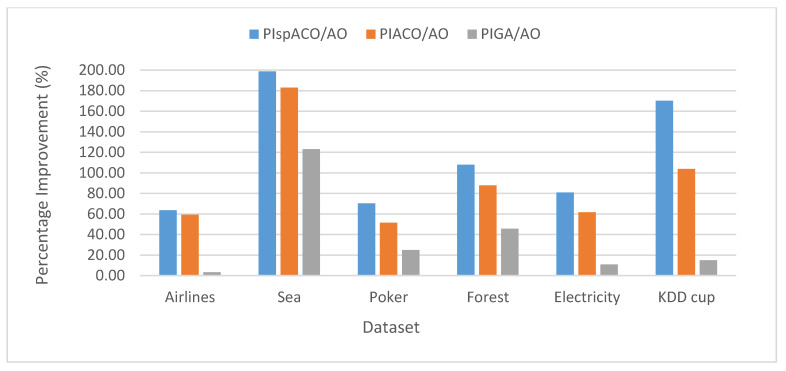
Analysis of PI for SC in different algorithms over IT2FCM-AO.

**Figure 3:**
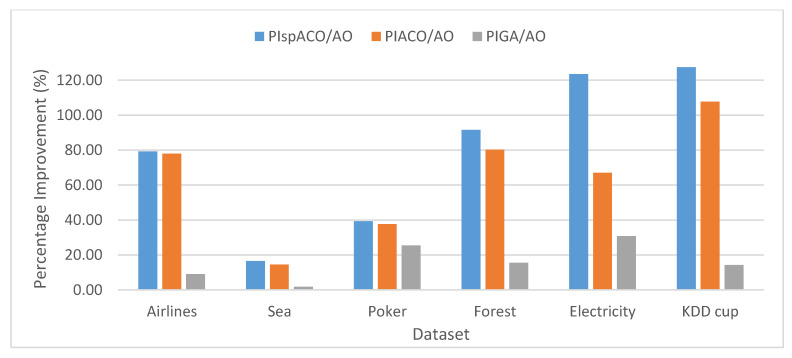
Analysis of PI for PCAES in different algorithms over IT2FCM-AO.

The Percentage Improvement (PI) determines the increase in the performance of the algorithm and is calculated as the percentage difference between the two algorithms. For example, PI in spIT2FCM-ACO over IT2FCM-AO (PIspACO/AO) is calculated as follows: $PIspACO/AO=\frac{SC_{spIT2FCM-ACO}-SC_{IT2FCM-AO}}{SC_{IT2FCM-AO}}*100$. Similarly, PI values can be calculated in different algorithms for other validity index measures. Figure 2 represents the analysis of PI in spIT2FCM-ACO (PIspACO/AO), IT2FCM-ACO (PIACO/AO), and GAIT2FCM (PIGA/AO) over IT2FCM-AO. It is found from the graph that the improvement in spIT2FCM-ACO over IT2FCM-AO is more than GAIT2FCM and IT2FCM-ACO for all the datasets. This indicates an improvement in the quality of the clusters generated by spIT2FCM-ACO compared with other algorithms. A similar observation is made in Figure 3 which presents PI in spIT2FCM-ACO (PIspACO/GA) and IT2FCM-ACO (PIACO/GA) over GAIT2FCM. Thus, among all the algorithms the improvement in GAIT2FCM over IT2FCM-AO shows lesser improvement. Thus, the improvement in GAIT2FCM over IT2FCM-AO indicates low performance across all the algorithms. This is because for large datasets GA has the tendency to get trapped in the local optimum solution.

A validity measure proposed by Wu and Yang [42] called Partial Coefficient and Exponential Separation (PCAES) is used to determine the clustering quality of the proposed algorithm. It is also based on fuzzy compactness and separation. The larger the value indicates each of the clusters is condensed and very much isolated from different clusters. It is computed as follows

(23) $$V_{PCAES}=\overset{c}{\underset{i=1}{\sum }}\overset{n}{\underset{j=1}{\sum }}u_{ij}^{2}/u_{M}-\overset{c}{\underset{i=1}{\sum }}exp\left(-\underset{k\neq i}{\min}\left\{\|v_{i}-v_{k}\|{}^{2}/\beta_{T}\right\}\right)$$

where, $u_{M}=\underset{1\leq i\leq c}{\min}\left(\overset{n}{\underset{j=1}{\sum }}u_{ij}^{2}\right),\;\beta_{T}=\overset{c}{\underset{l=1}{\sum }}\|v_{l}-\bar{v}{\|}^{2}/c,\;\bar{v}=\overset{n}{\underset{j=1}{\sum }}x_{j}/n$, $v_{i}$ center of each cluster, *c* number of clusters, $u_{ij}$ membership degree of data $x_{j}$ to cluster *i* and n size of dataset. 

Table 4 summarizes the results of PCAES for different algorithms along with SD. On comparing GAIT2FCM with IT2FCM-AO, an increase in performance is shown for all datasets. However, IT2FCM-ACO shows significant improvement over IT2FCM-AO for all the datasets. The increase is noteworthy compared to improvement in GAIT2FCM over IT2FCM-AO. This is because IT2FCM-ACO generates optimal cluster centers and avoids premature convergence [15]. Since PCAES is based on the structure of data, if obtained cluster centers are not optimal it would result in excessive overlapped clusters.

Similar to SC index, PCAES shows enhancement for IT2FCM-ACO when learned incrementally using single-pass approach. Since both cluster validity index measures the compactness and separation between the clusters using Euclidean distance and for large datasets it may give undesirable results. This is because for large datasets the ratio of the distance between the nearest and the farthest data point approaches to 1 i.e., the data points become uniformly distant from each other [43]. Since the basic concept is the closer points are more relevant than farthest point, however, if all the points are essentially uniformly distant from each other, the distinction is meaningless. Thus, grouping data points based on Euclidean distance for large datasets may result in poor clustering. Therefore, the clustering further improves using an incremental approach since Euclidean distance is calculated on small datasets. As a result, its performance improves and partition similar data into clusters.

Figure 3 represents the analysis of PI in spIT2FCM-ACO, IT2FCM-ACO and GAIT2FCM over IT2FCM-AO. The graph shows that the PI in spITT2FCM-ACO is high over IT2FCM-AO compared to other algorithms. Thus, the results obtained for SC and PCAES prove that the proposed algorithm spIT2FCM-ACO partitions large data more appropriately into clusters. In the following section, the accuracy of generated clusters is analyzed through external validity measures.

#### 5.3.2. External Criterion of Quality

External validity indexes measure the similarity between two clustering results of the same datasets [44]. The clustering quality is evaluated by estimating that the degree obtained for the generated clusters is similar to classes for a given dataset (classes must be known). Fuzzy Rand Index (FRI) [45] determines accuracy i.e., how accurately the given data points are divided into proper clusters. FRI is measured as follows

(24) FRI=a+d/a+b+c+d

For given two membership matrices ($U_{1},\;U_{2}$) a, b, c, d is defined as

$$a=\left|V\cap Y\right|=\sum_{j_{2}}^{N}\sum_{j_{1}}^{j_{2}-1}t\left(V\left(j_{1},j_{2}\right),Y\left(j_{1},j_{2}\right)\right);b=\left|V\cap Z\right|=\;\sum_{j_{2}}^{N}\sum_{j_{1}}^{j_{2}-1}t\left(V\left(j_{1},j_{2}\right),Z\left(j_{1},j_{2}\right)\right);$$

$$c=\left|X\cap Y\right|=\sum_{j_{2}}^{N}\sum_{j_{1}}^{j_{2}-1}t\left(X\left(j_{1},j_{2}\right),Y\left(j_{1},j_{2}\right)\right);d=\left|X\cap Z\right|\;=\sum_{j_{2}}^{N}\sum_{j_{1}}^{j_{2}-1}t\left(X\left(j_{1},j_{2}\right),Z\left(j_{1},j_{2}\right)\right)$$

where, $V\left(j_{1},j_{2}\right)$ is set of pairs of data objects belonging to same class in $U_{1}$; $X\left(j_{1},j_{2}\right)$ is set of pairs of data objects belonging to different class in $U_{1};\;Y\left(j_{1},j_{2}\right)$ is set of pairs of data objects belonging to same class in $U_{2}$; $Z\left(j_{1},j_{2}\right)$ is set of pairs of data objects belonging to different class in $U_{2}$ and *i* represents minimum t-norm.

**Figure 4:**
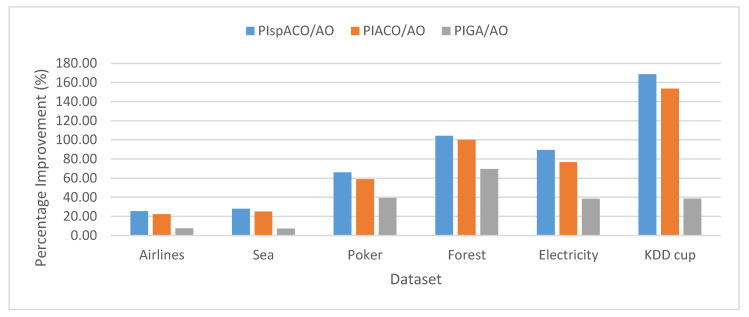
Analysis of PI for FRI in different algorithms over IT2FCM-AO.

Table 5 shows the results obtained for FRI using different algorithms and the SD. The high value of FRI for IT2FCM-ACO signifies increased clustering accuracy. Though, GA solves the issue of local optima and shows an improvement over IT2FCM-AO. However, for large datasets ACO alleviates the problem of local optima in IT2FCM more efficiently than GA. This is because for large datasets GA does not guarantee global optima solution. Moreover, with the increase in search space, it is sensitive to parameter settings that affect their performance. GA is also not suitable for dynamic datasets and has difficulty in solving constraint optimization problems [46]. The clustering quality is further improved for spIT2FCM-ACO compared to all the algorithms. It is found that as the size of the data is increasing the quality of the clusters generated is also increasing. Meanwhile, the accuracy achieved by IT2FCM-AO algorithm reduces with the increase in the size of the data. This is because as the data size is increasing the probability of getting stuck in local minima is also increasing which results in an inappropriate partition of data points. However, the proposed algorithm overcomes the issue of local optima and results in higher clustering accuracy. The comparison of PI in spIT2FCM-ACO in comparison to other algorithms is shown graphically in Figure 4. The graph shows the improvement in the proposed algorithm is higher than other algorithms. 

Error rate (ER) is another external criterion, which is defined as the number of data points allocated to an incorrect cluster. Higher the value of error rate indicates poor clustering quality while low values of error rate indicate good cluster quality generated by the algorithm in comparison to other algorithms.

**Figure 5:**
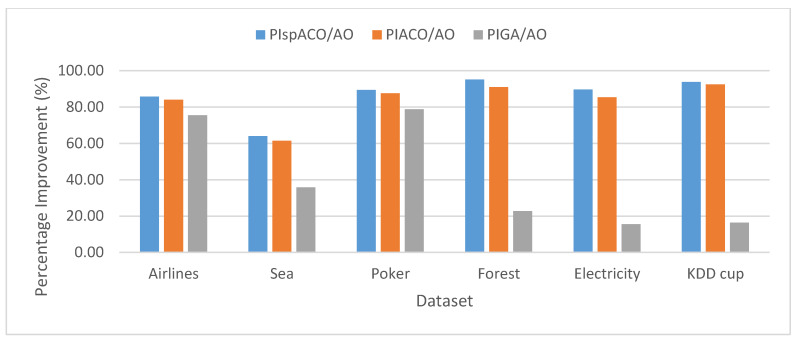
Analysis of PI for ER in different algorithms over IT2FCM-AO.

Table 6 defines the error rate for each algorithm. Low values of ER obtained for spIT2FCM-ACO compared to IT2FCM-AO, GAIT2FCM and IT2FCM-ACO show less error i.e., lesser number of data points allotted to the inappropriate cluster. PI in ER indicates a percentage decrease. Higher values of PI in spIT2FCM-ACO shown in Figure 5 suggests the decrease in the value of ER. For example, the error rate in spIT2FCM-ACO has reduced by 85% compared to IT2FCM-AO for airlines dataset.

#### 5.3.3. Simulation Analysis of Algorithm Computational Efficiency

This section presents the analysis of algorithm efficiency in terms of practical run time and speedup [47]. Run time for the algorithms is computed as the total amount of time spent to execute the main function and its child functions. Speedup measures the relative performance of two algorithms and is determined in terms of practical run time. Technically it is the improvement in the speed of algorithm in executing a similar task. The efficiency of the algorithm is improved in terms of reduced run time and increased speedup. Table 7 presents the comparison of run time for different algorithms for all the datasets. It is found that the run time of IT2FCM-ACO has increased compared to both IT2FCM-AO and GAIT2FCM algorithms. This signifies that IT2FCM-AO and GAIT2FCM converge faster than IT2FCM-ACO. To solve the issue of high convergence single-pass method is employed to IT2FCM-ACO. The results obtained from Table 7 verifies that with single-pass approach implemented in IT2FCM-ACO, the run time has improved significantly. spIT2FCM-ACO execute faster not only in comparison to IT2FCM-ACO but also to IT2FCM-AO and GAIT2FCM.

Though, airlines and forest datasets contain an almost equal number of instances but the run time for forest dataset is very high compared to airlines for all the algorithms. The reason for the difference in the two dataset lies in the number of variables. Forest dataset contains 54 attributes while airlines contain seven attributes. The same observation is made for the sea and poker datasets. Similarly, the poker and airline datasets have approximately the same number of variables but the number of instances in poker is nearly twice as airlines. So, longer run time is reported for poker dataset for all the algorithms. The run time for electricity and KDD cup datasets have increased substantially compared to other datasets. This is probably due to increase in the size of datasets. The size of the electricity dataset has increased significantly in terms of number of examples while KDD cup size has increased with respect to number of attributes in comparison to other datasets. Thus, the size of the dimension in terms of number of attributes and number of instances have an important influence on the run time of algorithms.

Table 8 presents the speed up results for different algorithms. SUAO/ACO represents speedup as the ratio of run time of IT2FCM-ACO and run time of IT2FCM-AO, SUspACO/AO denotes ratio of run time of IT2FCM-AO and run time of spIT2FCM-ACO, SUspACO/GA represents ratio of run time of GAIT2FCM and run time of spIT2FCM-ACO and SUspACO/ACO presents the ratio of run time of IT2FCM-ACO and run time of spIT2FCM-ACO. From Table 8 we can conclude that spIT2FCM-ACO execute faster not only in comparison to IT2FCM-ACO but also to IT2FCM-AO and GAIT2FCM. In the case of the sea dataset spIT2FCM-ACO is 3.21 faster than IT2FCM-AO and 4.69 times faster than IT2FCM-ACO. For large datasets airlines, forest, poker, electricity and KDD cup spIT2FCM-ACO is found to be approximately one to two times faster than IT2FCM-AO, GAIT2FCM and IT2FCM-ACO. The speed compared to all the algorithms has improved since, spIT2FCM-ACO works incrementally and thus, reduces the time required for clustering complete dataset. Therefore, the proposed algorithm spIT2FCM-ACO improves the efficiency of the algorithm IT2FCM-AO and shows significant improvement over GAIT2FCM and IT2FCM-ACO as well.

**Figure 6:**
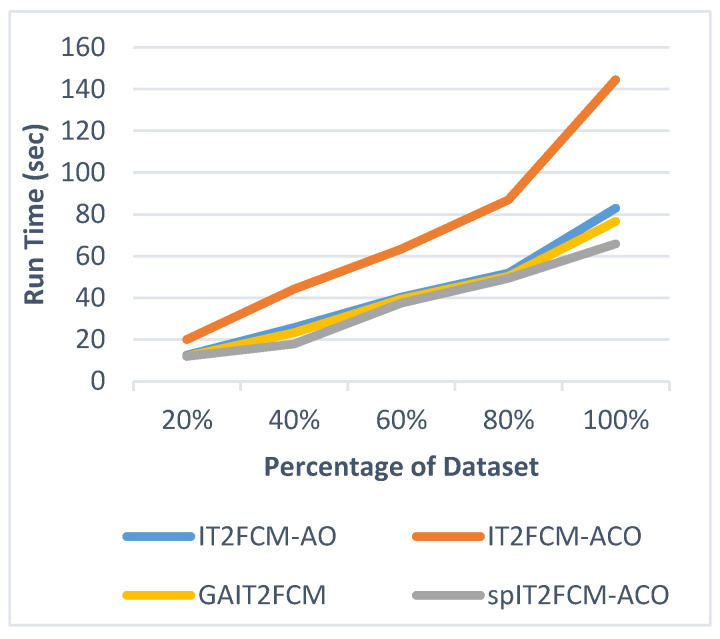
Analysis of run time for different percentages of airlines dataset.

**Figure 7:**
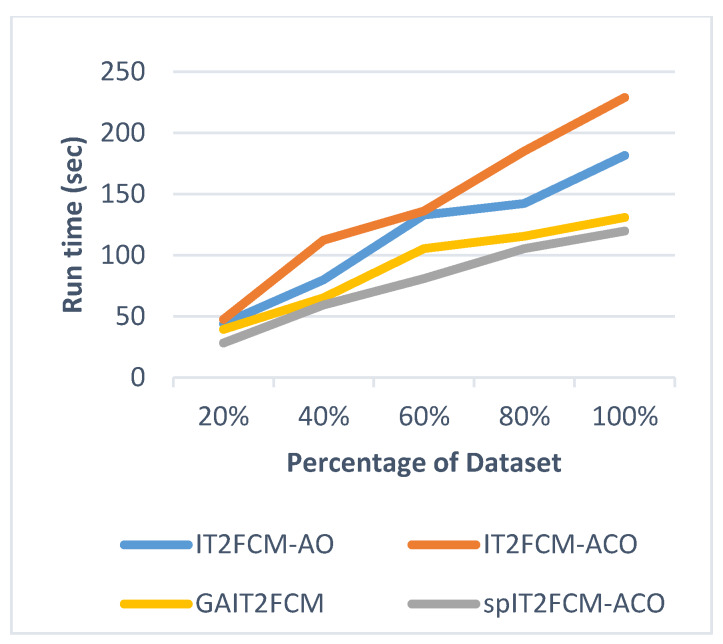
Analysis of run time for different percentages of forest dataset.

**Figure 8:**
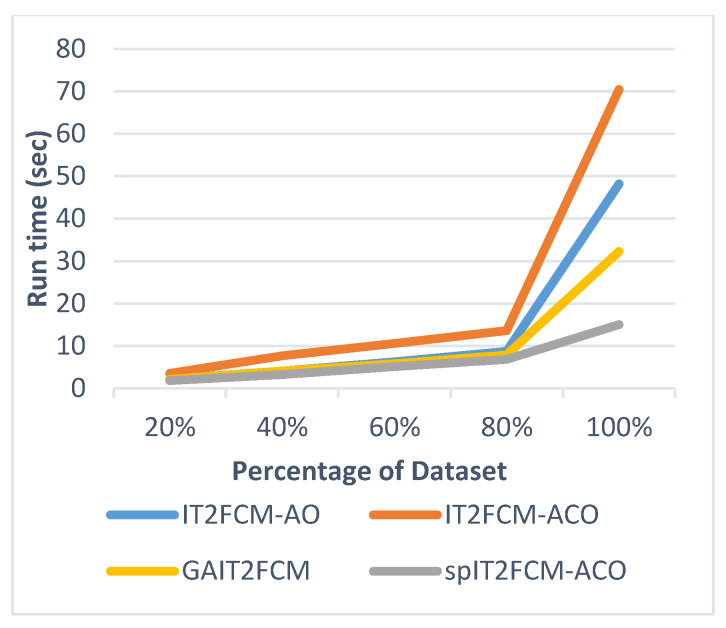
Analysis of run time for different percentages of sea dataset.

**Figure 9:**
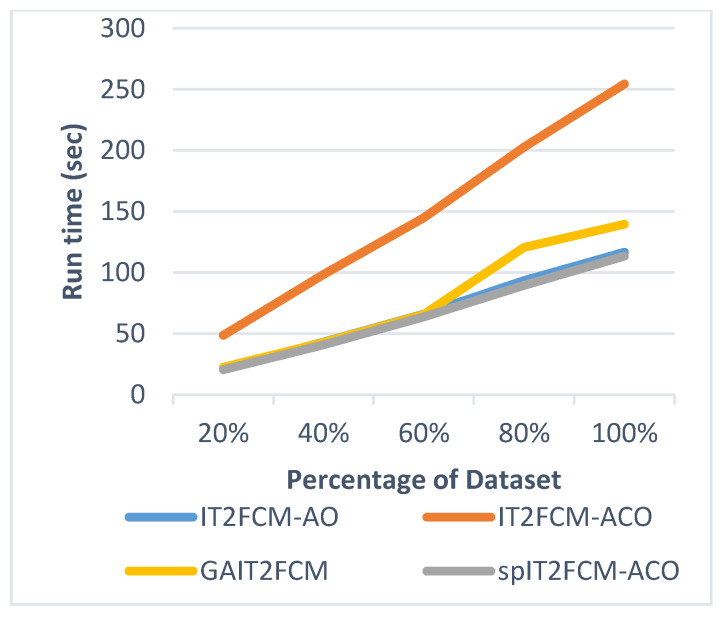
Analysis of run time for different percentages of poker dataset.

**Figure 10:**
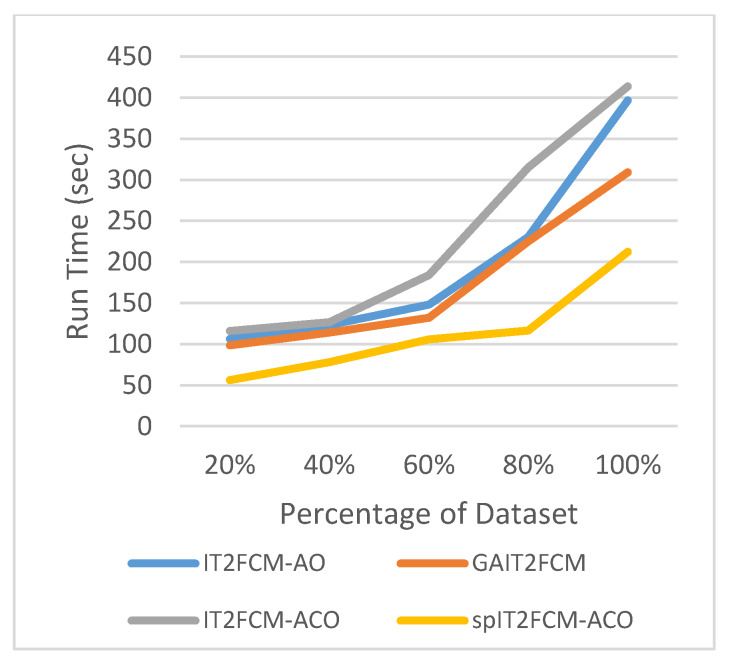
Analysis of run time for different percentages of electricity dataset.

**Figure 11:**
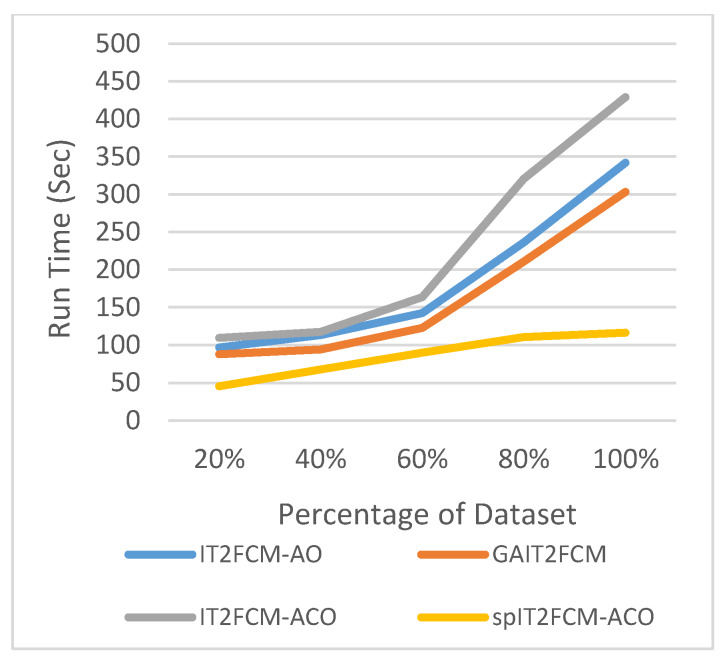
Analysis of run time for different percentages of KDD cup dataset.

The graphs illustrated by Figure 6, Figure 7, Figure 8, Figure 9, Figure 10 and Figure 11 compares the run time of IT2FCM-AO, GAIT2FCM, IT2FCM-ACO, and spIT2FCM-ACO for different percentages of datasets. Figure 6 gives results for airlines dataset where spIT2FCM-ACO is fastest for different percentage of dataset compared to the other algorithms. Similar results are found for sea, forest, poker, electricity, and KDD cup datasets. At 100% dataset a significant improvement in run time is observed for the proposed algorithm for all the datasets. This shows significance of the proposed algorithm specifically for large datasets.

### 5.4. Statistical Test

To draw a statistically meaningful inference about the performance of the proposed clustering algorithm, the numerical findings are further justified by conducting statistical tests. The Friedman statistical test is carried out to compare numerous algorithms [48]. It is a non-parametric test that has been used commonly for statistical comparison of multiple algorithms. Table 9 analyses the algorithm rankings in which spIT2FCM-ACO shows better performance than the other three over three measures: Fuzzy Rand Index (FRI), Error Rate (ER), and Run Time (RT).

The null hypothesis in the Friedman test states that all the clustering algorithms perform equally well for a given level. The Friedman statistic is given by Equation (25) where k is the number of algorithms, N is the total dataset, and R is the average rank of algorithms.

(25) $$\chi^{2}=\frac{12N}{k\left(k+1\right)}\left[\sum R^{2}-\frac{k{\left(k+1\right)}^{2}}{4}\right]$$

At a given value of α = 0.1 with three degrees of freedom, the critical value is determined to be 6.251. The value of $\chi^{2}$ for the three measures is found to be 18, 21.20, 16.98 and thus we can reject the null hypothesis. The main objective of the Friedman statistical test is to disclose the difference in performance among the algorithms. The rejection of null hypothesis proves that there is a significant difference in performance among clustering algorithms. However, this test does not prove that the spIT2FCM-ACO surpasses other algorithms statistically. This problem is solved by using Bonferroni–Dunn posthoc test [48], where spIT2FCM-ACO is better than other clustering algorithms if the difference in the performance is greater than the critical difference (CD). The CD is computed using Equation (26) where $q_{\alpha }$ gives critical values for posthoc tests.

(26) $$CD=q_{\alpha }\sqrt{\frac{k\left(k+1\right)}{6N}}$$

At α = 0.1 and *k* = 4, $q_{\alpha }$ is found to be 2.128 from the Bonferroni test critical table [48]. Thus, the value of CD is calculated to be 1.59. The performance of spIT2FCM-ACO in comparison to other algorithms is evaluated using Equation (27) where $R_{i},\;R_{j}$ represents the average rank of clustering algorithm *I* and *j* respectively. The value obtained (*z*) is used to evaluate the performance difference.

(27) $$z=\left(R_{i}-R_{j}\right)/\sqrt{\frac{k\left(k+1\right)}{6N}}$$

From Table 10 we can conclude that spIT2FCM-ACO shows superior performance over IT2FCM-AO and GAIT2FCM in the category of FRI and ER. However, with respect to IT2FCM-ACO it is below the critical difference, but close to it. On the other hand, spIT2FCM-ACO outperforms IT2FCM-AO and IT2FCM-ACO in run time while showing a slight reduction for GAIT2FCM. Thus, from the results, it can be interpreted that the incremental learning approach to IT2FCM-ACO performs well to reduce the run time and improve the accuracy and error rate of the algorithms.

## 6. Conclusions

This paper presents an improved spIT2FCM-ACO algorithm for large data streams. The proposed method solves the issue of continuous incoming data that poses time and memory restrictions by clustering each new incoming data incrementally. With reference to SC and PCAES fuzzy validity indices, it has been established that spIT2FCM-ACO generates good quality clusters. The FRI and ER values validate the performance of the proposed algorithm against other algorithms by achieving higher accuracy with fewer errors. The big-O computational analysis proves that spIT2FCM-ACO reduces time and memory constraints through incremental learning compared to IT2FCM-ACO. Further, the efficiency of the algorithms is analyzed in terms of time and speedup. From the results, it is found that for large datasets IT2FCM-ACO takes a significant amount of run time to execute. However, when IT2FCM-ACO is executed with the single-pass technique the run time reduces substantially. Thus, the decrease in run time and increase in speed up proves the significance of spIT2FCM-ACO over IT2FCM-AO, GAIT2FCM, and IT2FCM-ACO. However, the proposed method spIT2FCM-ACO has only been tested for large datasets. In the future the authors recommend analyzing spIT2FCM-ACO on unloadable data i.e., very large and huge data to model the characteristics of big data.

## Figures and Tables

**Table 1:** Parameter initialization for spIT2FCM-ACO.

Parameter	Value
Maximum iteration $\left(t_{max}\right)$	1000
Fuzzifiers (*m_1_*)	1.7
Fuzzifier (*m_2_*)	2.6
Termination condition (min_impro)	1*10^−5^
Pheromone evaporation rate (ρ)	0.05
Parameter to avoid division by 0 (ε)	0.01
Varies speed of convergence (α)	1.0

**Table 2:** A summary of Dataset.

Dataset	# attr	# eg	# Class
Airlines [34]	7	539,384	2
Forest [35]	54	581,012	7
Sea [36]	3	1,000,001	2
Poker [37]	10	1,025,010	10
Electricity [35]	9	2,075,259	2
KDD cup [34]	41	494020	23

**Table 3:** Evaluation of SC index.

Dataset	Airlines	Sea	Poker	Forest	Electricity	KDD Cup
IT2FCM-AO	1.57 ± 1.01	0.82 ± 0.35	2.12 ± 0.48	2.28 ± 1.21	1.20 ± 1.21	1.07 ± 0.21
GAIT2FCM	1.62 ± 0.34	1.83 ± 0.29	2.65 ± 0.18	3.32 ± 0.18	1.33 ± 1.01	1.23 ± 0.15
IT2FCM-ACO	2.5 ± 0.04	2.32 ± 0.11	3.21 ± 0.05	4.28 ± 0.11	1.94 ± 0.52	2.18 ± 0.09
spIT2FCM-ACO	2.57 ± 0.01	2.45 ± 0.02	3.61 ± 0.03	4.74 ± 0.02	2.17 ± 0.08	2.89 ± 0.06

**Table 4:** Evaluation of PCAES index.

Dataset	Airlines	Sea	Poker	Forest	Electricity	KDD Cup
IT2FCM-AO	1.54 ± 0.33	7.25 ± 0.21	5.02 ± 0.19	1.42 ± 1.20	0.94 ± 0.75	1.68 ± 0.92
GAIT2FCM	1.68 ± 0.58	7.38 ± 0.96	6.30 ± 0.17	1.64 ± 1.08	1.23 ± 0.4	1.92 ± 0.51
IT2FCM-ACO	2.74 ± 0.09	8.30 ± 0.47	6.91 ± 0.02	2.56 ± 1.04	1.57 ± 0.34	3.49 ± 0.20
spIT2FCM-ACO	2.76 ± 0.06	8.45 ± 0.07	6.99 ± 0.01	2.72 ± 1.02	2.10 ± 0.32	3.82 ± 0.10

**Table 5:** Evaluation of FRI.

Dataset	Sea	Airlines	Poker	Forest	Electricity	KDD Cup
IT2FCM-AO	0.67 ± 0.12	0.68 ± 0.05	0.56 ± 0.03	0.46 ± 0.17	0.47 ± 0.15	0.53 ± 0.11
GAIT2FCM	0.72 ± 0.07	0.73 ± 0.04	0.78 ± 0.03	0.78 ± 0.13	0.65 ± 0.13	0.72 ± 0.10
IT2FCM-ACO	0.82 ± 0.02	0.85 ± 0.02	0.89 ± 0.01	0.92 ± 0.12	0.83 ± 0.10	0.88 ± 0.04
spIT2FCM-ACO	0.84 ± 0.01	0.87 ± 0.01	0.93 ± 0.001	0.94 ± 0.11	0.89 ± 0.05	0.91 ± 0.001

**Table 6:** Evaluation of ER.

Dataset	Airlines	Sea	Poker	Forest	Electricity	KDD Cup
IT2FCM-AO	1.19 ± 0.21	0.39 ± 0.05	1.13 ± 0.13	1.45 ± 1.10	1.16 ± 0.03	1.46 ± 0.24
GAIT2FCM	0.29 ± 0.12	0.25 ± 0.03	0.24 ± 0.09	1.12 ± 0.90	0.98 ± 0.13	1.22 ± 0.11
IT2FCM-ACO	0.19 ± 0.52	0.15 ± 0.002	0.14 ± 0.05	0.13 ± 0.30	0.17 ± 0.13	0.11 ± 0.06
spIT2FCM-ACO	0.17 ± 0.03	0.14 ± 0.001	0.12 ± 0.03	0.07 ± 0.23	0.12 ± 0.10	0.11 ± 0.03

**Table 7:** Evaluation of Run Time (s).

Dataset	Airlines	Sea	Poker	Forest	Electricity	KDD Cup
IT2FCM-AO	82.18 ± 1.21	48.18 ± 0.21	116.60 ± 0.36	181.69 ± 1.68	397.06 ± 1.23	341.90 ± 0.98
GAIT2FCM	76.67 ± 0.78	32.25 ± 0.12	139.67 ± 0.24	130.95 ± 1.42	309.27 ± 1.10	303.14 ± 0.27
IT2FCM-ACO	144.54 ± 0.50	70.45 ± 0.04	254.38 ± 0.08	229.13 ± 1.35	413.94 ± 0.78	428.69 ± 0.02
spIT2FCM-ACO	65.84 ± 0.003	15.03 ± 0.004	105.49 ± 0.06	119.68 ± 0.07	212.67 ± 0.03	116.24 ± 0.004

**Table 8:** Evaluation of Speed Up.

Dataset	SspACO/AO	SspACO/GA	SspACO/ACO
Sea	3.21	2.15	4.69
Airlines	1.25	1.16	2.19
Forest	1.51	1.16	1.91
Poker	1.10	1.24	2.41
Electricity	1.87	1.45	1.95
KDD cup	2.94	2.60	3.68

**Table 9:** Ranking of Algorithms based on FRI, ER, and RT.

Dataset	Rank (FRI, ER, RT)			
	IT2FCM-AO	GAIT2FCM	IT2FCM-ACO	spIT2FCM-ACO
Airlines	(4, 4, 3)	(3, 3, 2)	(2, 2, 4)	(1, 1, 1)
Sea	(4, 4, 3)	(3, 3, 2)	(2, 2, 4)	(1, 1, 1)
Forest	(4, 4, 3)	(3, 3, 2)	(2, 2, 4)	(1, 1, 1)
Poker	(4, 4, 2)	(3, 3, 3)	(2, 2, 4)	(1, 1, 1)
Electricity	(4, 4, 3)	(3, 3, 2)	(2, 2, 4)	(1, 1, 1)
KDD cup	(4, 4, 3)	(3, 3, 2)	(2, 2.5, 4)	(1, 2.5, 1)
Average Rank	(4, 4, 2.83)	(3, 3, 2.17)	(2, 2.08, 4)	(1, 1.25, 1)

**Table 10:** Evaluation of performance difference between spIT2FCM-ACO and other algorithms.

Algorithms	z Value		
	FRI	ER	RT
spIT2FCM-ACO vs. IT2FCM-AO	4.02	3.69	2.45
spIT2FCM-ACO vs. GAIT2FCM	2.68	2.35	1.57
spIT2FCMACO vs. IT2FCM-ACO	1.34	1.45	4.02
